# Exploration of spatiotemporal heterogeneity and socio-demographic determinants on COVID-19 incidence rates in Sarawak, Malaysia

**DOI:** 10.1186/s12889-023-16300-8

**Published:** 2023-07-20

**Authors:** Piau Phang, Jane Labadin, Jamaludin Suhaila, Saira Aslam, Helmy Hazmi

**Affiliations:** 1 Faculty of Computer Science and Information Technology, Universiti Malaysia Sarawak, Kota Samarahan, 94300 Sarawak, Malaysia; 2 Department of Mathematical Science, Faculty of Science, Universiti Teknologi Malaysia, Skudai, 81310 Johor, Malaysia; 3 Faculty of Medicine and Health Science, Universiti Malaysia Sarawak, Kota Samarahan, 94300 Sarawak, Malaysia

**Keywords:** COVID-19, Spatiotemporal heterogeneity, Socio-demography, Spatial lag, Spatial error model, Geographically weighted regression

## Abstract

**Background:**

In Sarawak, 252 300 coronavirus disease 2019 (COVID-19) cases have been recorded with 1 619 fatalities in 2021, compared to only 1 117 cases in 2020. Since Sarawak is geographically separated from Peninsular Malaysia and half of its population resides in rural districts where medical resources are limited, the analysis of spatiotemporal heterogeneity of disease incidence rates and their relationship with socio-demographic factors are crucial in understanding the spread of the disease in Sarawak.

**Methods:**

The spatial dependence of district-wise incidence rates is investigated using spatial autocorrelation analysis with two orders of contiguity weights for various pandemic waves. Nine determinants are chosen from 14 covariates of socio-demographic factors via elastic net regression and recursive partitioning. The relationships between incidence rates and socio-demographic factors are examined using ordinary least squares, spatial lag and spatial error models, and geographically weighted regression.

**Results:**

In the first 8 months of 2021, COVID-19 severely affected Sarawak’s central region, which was followed by the southern region in the next 2 months. In the third wave, based on second-order spatial weights, the incidence rate in a district is most strongly influenced by its neighboring districts’ rate, although the variance of incidence rates is best explained by local regression coefficient estimates of socio-demographic factors in the first wave. It is discovered that the percentage of households with garbage collection facilities, population density and the proportion of male in the population are positively associated with the increase in COVID-19 incidence rates.

**Conclusion:**

This research provides useful insights for the State Government and public health authorities to critically incorporate socio-demographic characteristics of local communities into evidence-based decision-making for altering disease monitoring and response plans. Policymakers can make well-informed judgments and implement targeted interventions by having an in-depth understanding of the spatial patterns and relationships between COVID-19 incidence rates and socio-demographic characteristics. This will effectively help in mitigating the spread of the disease.

## Background

**Fig. 1:**
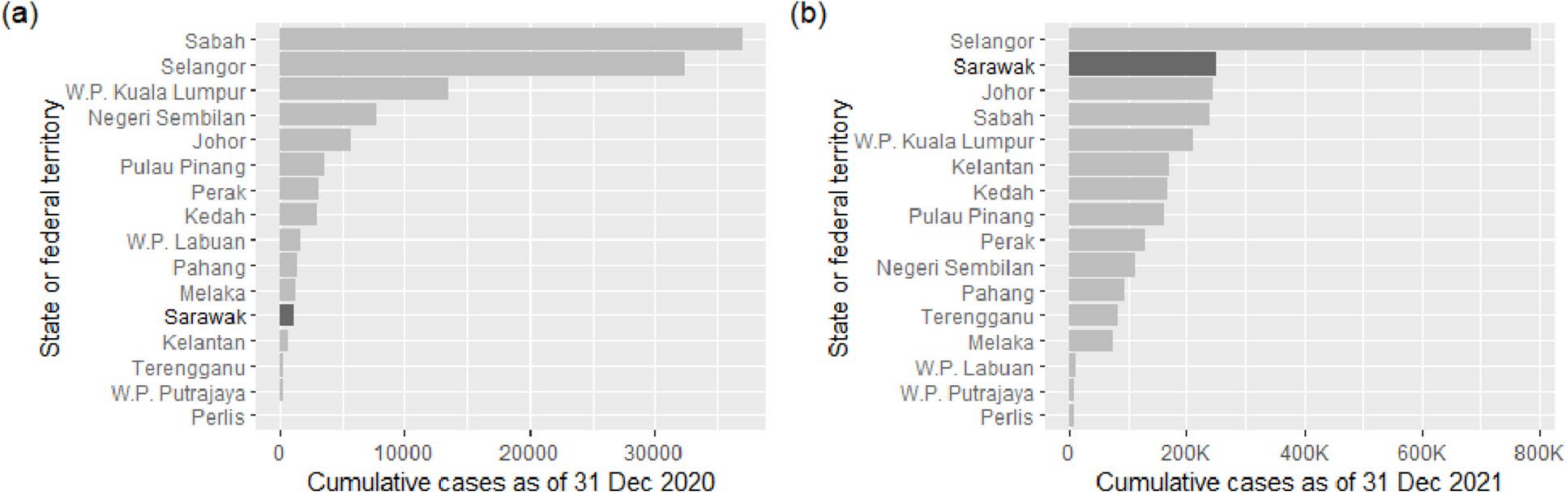
The graph of cumulative cases among 13 states and 3 federal territories in Malaysia as of (**a**) 31 Dec 2020, (**b**) 31 Dec 2021

The coronavirus disease 2019 (COVID-19), which first surfaced at the end of 2019, has rapidly spread to become a global public health issue. Over 650 million verified cases and over 6 million fatalities had been reported worldwide, as of 23 December 2022. In contrast, there have been more than 5 million cases and 36 000 deaths overall in Malaysia [1], which has a population of 32.78 million. In 2020, COVID-19 could be considered well-controlled in the state of Sarawak, Malaysia. Nevertheless, the easing of border controls across state lines following a state election in Sabah at the end of the third quarter of 2020 accelerated the disease spillover and propagation in all states of Malaysia [2, 3], and Sarawak is no exception. Sarawak had the second highest cumulative case count among Malaysia’s 13 states and three federal territories at the end of 2021, with more than 250 000 and 1 619 fatalities (see Fig. 1).

COVID-19 continues to present a serious and changing threat to the world. Despite the optimism that has been generated by the availability of various vaccinations, the virus continues to decimate communities worldwide. Since the beginning of 2021, several varieties of the virus have evolved and taken over in many nations, with Alpha, Beta, Gamma, Delta, and Omicron variants being the most virulent. The World Health Organization classified the Delta and Omicron variants as variants of concern on May 11, 2021, and November 26, 2021, respectively. These two variants have been widely circulating. Omicron had several mutations which negatively altered the COVID-19 epidemiology [4]. The COVID-19 pandemic incidence-related worldwide crisis was the most important troublesome event in recent history [5]. Both the global economy and each citizen's day-to-day activities are significantly impacted. A number of businesses have suffered grave consequences as a result of abrupt disruptive transitions. As preventive measures, people must develop new behavioral patterns including the use of face mask, hand sanitization, and physical distancing.

The 3-year coronavirus pandemic that has affected the entire world can be categorized chronologically into four stages: COVID Zero, mass vaccination, living with COVID-19, and the start of interpandemic periods. The spatial transmission of COVID-19 in mainland China and the regional pattern of virus importations across countries on a global scale are the focus of the research community in spatial epidemiology in the first half of the year 2020 [6–8]. This is then followed by more evidence of spatial autocorrelation of confirmed cases of COVID-19 on a local scale in the second half of the year 2020 [9, 10]. Subsequently, the spatial heterogeneity and the diffusion of COVID-19 infection on different spatial scales [11], either across or within a country [12], state, province [13], county [14, 15], district [16, 17], and city [18] have become the key subjects in unfolding the outbreak.

COVID-19 is also a rapidly evolving pandemic with multiple peaks and waves. Consequently, the initial focus on spatial heterogeneity of the pandemic has substantially shifted to describing distinct temporal heterogeneity in COVID-19 transmission over time [19–24]. The multi-peaked characteristics may probably be attributable to structure within an area, geography, or numerous other factors [25]. These factors include but are not limited to environmental factors (climate, geographic location, and pollution), human activities (mobility, socio-economic, health, demographic, and intervention), and built environments (density, housing conditions, and basic amenities), as highlighted in previous studies [11, 26, 27]. Furthermore, the effects of these factors on COVID-19 transmission may be diverse or even contrasting spatially and temporally. That is, although certain factors might be positively associated in one spatial context or one scale, they could be insignificant or negative in another spatial context or scale [11].

At the district level of Sarawak, the progression of COVID-19 cases is anticipated to consistently exhibit spatiotemporal heterogeneity. Sarawak Disaster Management Committee (SDMC) has the responsibility to coordinate the management of the pandemic in the state of Sarawak. They are in charge of disseminating official news, maintaining information and carrying out the standard operating procedures (SOPs) of the preventive measures set by the federal government. Some of the SOPs have been modified to take into account the local Sarawak COVID-19 conditions. Given that 45.3% of Sarawak’s population lives in rural areas [28] with few medical services and resources, these regional autonomy regulations are crucial for the state of Sarawak.

The essential lesson of the pandemic, however, is the profound influence that structural disadvantage and inequality have on its course and outcomes [29]. Through ecological research, such structural disadvantage indicators during a pandemic could be well explored. As the finest spatial scale of COVID-19 and census data available in Sarawak is at the district level, an ecological study focusing solely on the relationship between socio-demographic determinants and COVID-19 confirmed cases for all 40 districts in Sarawak is crucial. Although several spatial modelling and regression studies had been conducted at the district level in Malaysia [30–32], they only investigated the diseases evolution until February 2021. Therefore, this present study not only extends our previous work [33] on Sarawak by analyzing four different temporal periods throughout 2021, but also considers several potential socio-demographic indicators suggested in previous work [31].

This study aims to investigate the association between district-wise disease incidence rates and socio-demographic indicators through global and local spatial regression over four different temporal periods. It offers several unique contributions not commonly found in the application of spatial statistics in the epidemiological literature.

First, we utilized change point analysis to subdivide the data into several temporal periods or waves. This approach allows for a more nuanced understanding of the disease dynamics over time and enables us to identify any significant shifts or changes in the spatial patterns of COVID-19 incidence rates. Second, we used regularization techniques and recursive partitioning in the variable selection process for our regression models. These methods help us identify the most relevant socio-demographic factors that contribute significantly to the spatial variation in COVID-19 incidence rates, thereby enhancing the accuracy and interpretability of our findings. Third, we went beyond the commonly used first-order spatial contiguity matrix and incorporated second-order spatial contiguity in our global spatial regression models. This consideration allows us to capture more complex spatial relationships and better account for potential spatial autocorrelation in the data.

Furthermore, we included a detailed flowchart summarizing our methodology to facilitate understanding, particularly for readers such as public health officials who may not be familiar with spatial statistics. We believe that this visual representation will assist them in comprehending the analytical process and interpreting the results effectively. This research provides references for the prevention and control of COVID-19 related infectious diseases as well as evidence for disease surveillance and response by using geographical information derived from spatial modelling.

The following sections show detailed methodology including a brief description of the study area, data source, and preprocessing of response and explanatory variables are first outlined prior to the presentation of the variable selections procedure and spatial regression modelling. Analysis of results, discussion, conclusion, limitations and opportunities are also given.

## Methods

### Study area

**Fig. 2:**
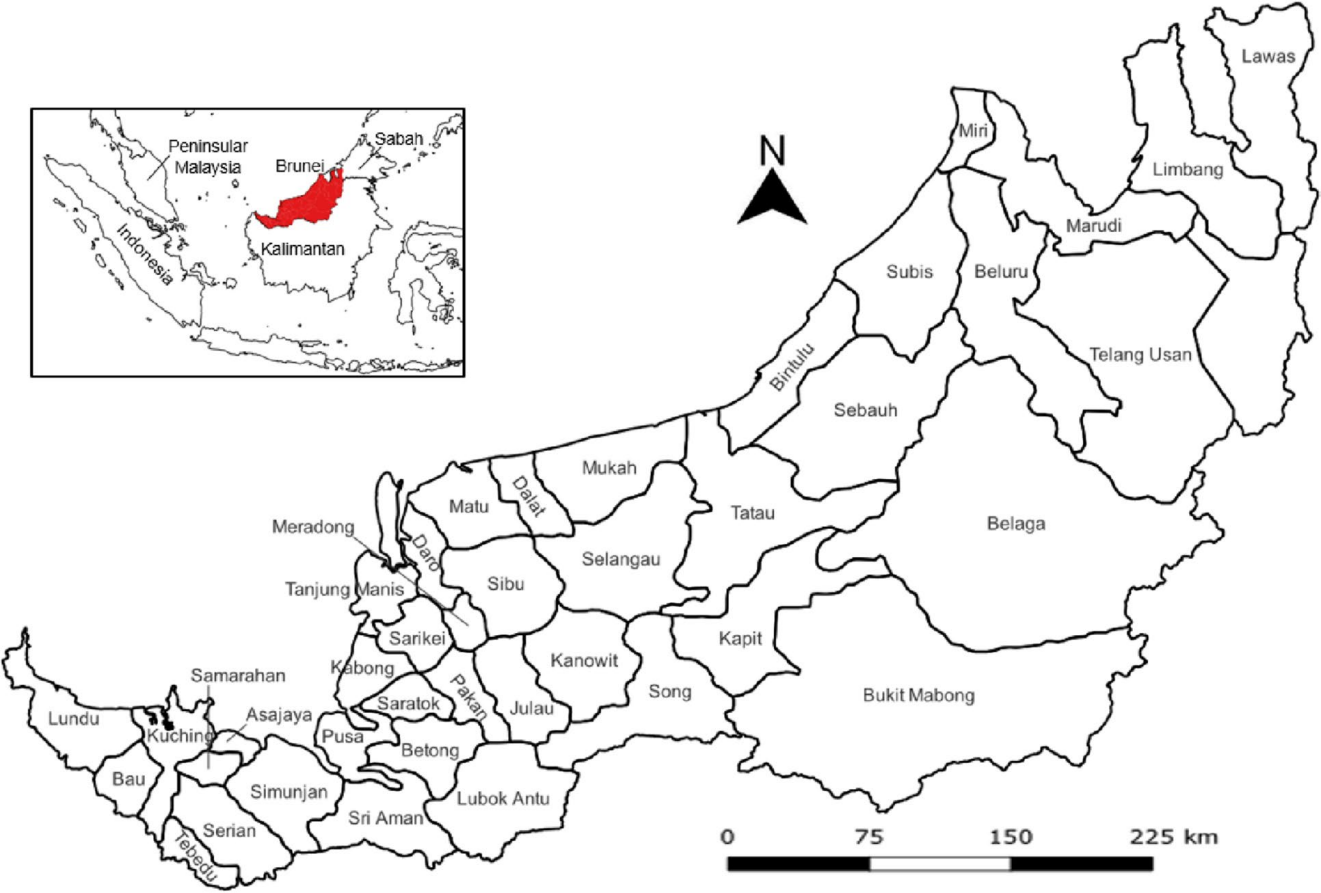
Map of 40 districts in Sarawak, Malaysia. Insert: Location of Sarawak (highlighted red) and its neighboring regions

Sarawak is the largest state in Malaysia by land area and is separated from Peninsular Malaysia by the South China Sea. Sarawak is divided into 12 administrate divisions and further subdivided into 40 districts (see Fig. 2), in which Kuching City is the capital of Sarawak. Sarawak shares an interstate border with Sabah via the Lawas district; national borders with Brunei via districts Miri, Marudi, Limbang and Lawas in the north; and Indonesia via the Sarawak − Kalimantan border. As of 2020, the population of Sarawak was estimated to be approximately 2.45 million, making the Sarawak population the fifth highest by state in Malaysia. Even so, Sarawak has the lowest population density with 20 persons per square kilometer. Nearly 57.5% of the Sarawak population resides in five urban districts, namely, Kuching, Samarahan, Sibu, Bintulu, and Miri [34].

### Data source

COVID-19 data were extracted from the daily press statements published by the Sarawak Disaster Management Committee (SDMC). The daily COVID-19 confirmed cases from 1 January to 31 December 2021, are collected for all 40 districts in Sarawak. The administrative boundary shapefile of Sarawak districts is obtained from an open license resource known as geoBoundaries Global Administrative Database [35]. Additional four other districts, namely, Telang Usan, Beluru, Subis, and Sebauh are included, and some minor modifications on the shapefile are performed using the open source geographic information system (GIS) application QGIS Desktop 3.24.3 [36].

Table 1 lists all 14 initially selected explanatory variables. The choice of these variables is based on the availability of data and localizing to Sarawak’s socio-demographic context. Although the list may not be exhaustive, all variables (except the first four and Median_inc) are indicators of social vulnerability in existing COVID-19 research, as mentioned in the review paper [37]. The first four variables and Median_inc are chosen since they are found to be significant in COVID-19 incidence rates in existing research [38–40], respectively. The household income and basic amenities data per administrative district in Sarawak are derived from the survey report released by the Department of Statistics Malaysia [34], whereas the Population and Housing Census of Malaysia 2020 provided the demographic data [28]. Environmental factors, transport, and mobility elements at the district level are not considered in this study due to data unavailability. Since the exploratory variables are in different orders of magnitude, the standardization procedure will take place before modelling.

**Table 1:** Description of explanatory variables

Category / Variable name	Abbreviation	Variable description
**Demographic**		
Population growth rate	Pop_GrowthR	The rate at which the number of residents increases (or decreases) compared to that in the previous year
Non-citizen population	Pop_NonCitizen	Percentage of residents who are non citizens
Male population	Pop_Male	Percentage of residents who are male
Population between 15 and 64	Pop_15to64	Percentage of residents who are between 15 and 64
Population 65 years and above	Pop_65	Percentage of residents who are 65 years and above
Population density	Pop_Density	The average number of residents per km^2^
Household size	Household_size	Average number of persons living together in a dwelling unit
**(Household) income-related criteria**		
Median of monthly income	Median_inc	Median of monthly household gross income
Gini coefficient	Gini	A measure for representing the income inequality
Incidence of poverty	Poverty	Proportion of households under the poverty line income
**Basic amenities / urban infrastructure**		
Internet subscription	Internet	Percentage of households owned subscription to the internet at home
Piped water	Piped_water	Percentage of households with piped water supply in the house
Public health centers	Public_h	Percentage of households with less than 5 km distance from living quarters to the nearest public health centers
Garbage collection facility	Garbage	Percentage of households with garbage collection facility (within 100 m away from living quarters)

### Data preprocessing for the response variable

This study accesses the evolution of Sarawak COVID-19 incidence rates and their relationship with socio-demographic indicators at the district level in different temporal periods. By keeping the COVID-19 data for the entire of 2021 as reference or baseline data, the number of COVID-19 daily confirmed cases in Sarawak for 2021 will be divided into four temporal periods based on change-point analysis. Change point analysis is used to identify times in a time series at which abrupt changes (for instance, mean shift and/or variance change) occur and is implemented using the changepoint package [41] in R programming. Then, the district-wise disease incidence rate for the respective temporal period is calculated using the Eq. (1):

1 $$\text{Incidence rate}=\frac{\text{The number of confirmed cases in a temporal period}}{\text{The population of a district}}\times 1000$$

The normality assumption of the response variable (incidence rate) for different periods is checked by plotting a histogram and performing the Shapiro–Wilk test. The Shapiro–Wilk test is a formal test for the normality of residual distribution. If the Shapiro–Wilk *p*-value is greater than 0.05, it suggests that the data are normally distributed. Otherwise, the logarithmic transformation will be performed on the response variables to approximately conform to normality.

### Variable selection for regression models

Both the regularization technique and recursive partitioning, as illustrated in detail in [42], are employed to identify the best subset of explanatory variables to include in the regression models. This variable selection procedure is first conducted by fitting a generalized linear model with regularized least squares using R package glmnet [43] for all 14 explanatory variables listed in Table 1 and the district-wise incidence rates for 2021 as a response variable. Regularization aims at preventing overfitting or underfitting in linear models by tuning a penalty term to the loss function and finding the optimal set of variables in the least squares method. In short, the alpha (α) value that regularizes the penalty term is being calibrated. Depending on the α value, three types of regularized least squares regressions can be established. The elastic net regression takes a value between ridge regression (α  = 0) and lasso regression (α  = 1).

After choosing an α value and obtaining the desired number of variables, a regression tree is fit by employing a recursive partitioning algorithm in the rpart package [44]. The variable importance of each explanatory variable obtained by recursive partitioning is plotted to determine which variables to be considered in the subsequent analysis.

### Spatial regression modelling

#### Spatial weights

The spatial association of the districts can be revealed through the construction of spatial weights based on either spatial contiguity or geographical distance. For spatial contiguity, both first- and second-order queen contiguity spatial weights will be used in the global spatial regression model, whereas the geographical distance will be employed in the local spatial regression model in this study. The spatial weight is specified in two ways to capture the varying natures of the district-wise spatial dependence and to ensure the robustness of our estimate.

The first-order queen contiguity spatial weight is defined in Eq. (2) as follows:

2a $${w}_{ij}{\prime}=\left\{\begin{array}{ll}1 &\text{if a district}\,j\,\text{shares a common boundary with another district}\,i\\ 0& \text{otherwise}\end{array}\right.$$

2b $${w}_{ij}=\frac{{w}_{ij}{\prime}}{{\sum }_{j=1}^{n}{w}_{ij}{\prime}}$$

where ${w}_{ij}$ is the normalized weight in which its magnitude indicates the intensity of spatial proximity between two districts and *n* = 40 in this study. Assumes that the district-wise incidence rates are influenced by the first-order neighbors and the second-order neighboring districts’ rates, which takes into account the neighbor of the neighbor. For instance, the Bau district has two first-order neighbors, that is, Kuching and Lundu. Still, it has another four second-order neighbors, namely, Asajaya, Samarahan, Serian and Tebedu, which are first-order neighbors of Kuching.

Both orders of queen contiguity spatial weights are constructed with R package spdep [45]. The average number of neighbors is 4.4 and 11.3, respectively, for first- and second-order contiguities. Sibu is the most connected district with eight neighbors in the first-order contiguity weights, whereas Julau and Selangau with 18 neighbors in the second-order contiguity. Since no standard scientific criterion exists for selecting an optimal spatial weight matrix or neighborhood structure [46], we will present a spatial autocorrelation analysis for district-wise incidence rates in different temporal periods obtained from both orders of spatial contiguity weights.

### Global spatial autocorrelation

The spatial autocorrelation analysis is then performed to determine to what extent the systematic spatial variation in district-wise incidence rates is presented. That is, the following global Moran’s *I* statistic (see Eq. 3) is calculated to investigate whether the close districts have the tendency to have similar incidence rates

3 $$I=\frac{n}{{S}_{0}}\frac{{\sum }_{i=1}^{n}{\sum }_{j=1}^{n}{w}_{ij}({x}_{i}-\overline{x })({x}_{j}-\overline{x })}{{\sum }_{i=1}^{n}{({x}_{i}-\overline{x })}^{2}}$$

In the formula above, *n* is the number of districts, ${S}_{0}$ is the aggregation of all the spatial weights, ${x}_{i}$ (resp. ${x}_{j}$) is the disease incidence rate at a particular (resp. another) district and $\overline{x }$ is the mean of the incidence rate.

### Local spatial autocorrelation

The local indicator of spatial association (LISA) cluster map is plotted to illustrate the type of spatial autocorrelation for each district with an assessment of the significant levels of local spatial statistics. Two broad categories of local spatial association exist: spatial cluster (positive local spatial autocorrelation) and spatial outlier (negative local spatial autocorrelation). A spatial cluster shows either high-high or low-low spatial association, whereas a spatial outlier consists of either high-low or low–high values. High-high (resp. low-low) clustering pattern refers to districts with high (resp. low) incidence rates surrounded by high (resp. low) incidence rate neighbors. Meanwhile, high-low (resp. low–high) outlier means that districts with high (resp. low) incidence rates are surrounded by low (resp. high) incidence rate neighbors. The disease spillover risk is higher in the districts identified as spatial outliers.

### Global spatial regression (SLM and SEM)

Both global and local spatial regression models are constructed to find the linear relationship between response variables (district-wise incidence rates) and explanatory variables by taking into account geographical information, especially the spatial dependence between districts. The two general ways to incorporate spatial dependence in global spatial regression models are through formulating a spatial lag model (SLM) and a spatial error model (SEM). In the SLM, the spatial dependence is explicitly incorporated by adding a spatially lagged dependent variable on the right-hand side of a linear regression equation, as given in Eq. (4).

4 $$y=X\beta +\rho Wy+\varepsilon$$

where *y* is the 1 × *n* vector of the response variable; *X* is the *n* × *k* matrix of the explanatory variable; *W* is the *n* × *n* spatial weight matrix defined in Eq. (2); *β* is the coefficients estimate of the explanatory variable; *ρ* is a scalar spatial autoregressive parameter (also known as a lag parameter) which indicates how much the incidence rate in districts is influenced by its neighboring districts; and *ε* is the random error.

SLM implies that a unit change of response variable in one district impacts other districts. In other words, the underlying assumption of SLM in this study is that the incidence rates in one district are directly influenced by the rates found in that district’s neighbors. Consequently, different spatial weights matrices may yield different results.

Only a few explanatory variables, however, are included in this analysis due to data availability constraints. It follows that spatial error might exist due to unmeasured variables that could be related through geographical proximity. Therefore, spatial patterns of this unexplained variation should be inspected by constructing a SEM, as given in Eq. (5).

$$y=X\beta +u$$

where

5 $$u=\lambda Wu+\varepsilon$$

where *λ* is the spatially correlated lag error parameter and $\lambda Wu$ is the spatial error term. If *λ* is statistically significant, it can be deduced that hidden explanatory variables with spatial autocorrelation exist, which may lead to non-negligible spatial autocorrelation in the residual.

### Local spatial regression (Geographically weighted regression)

Geographically weighted regression (GWR) is a local spatial regression model with variable coefficients (i.e., the regression coefficients are not fixed but depend on the geographical coordinate of observations). The GWR model is used to explore the non-stationary spatial relationships between COVID-19 incidence rates and socio-demographic factors based on the hypothesis of proximity correlation. It gives the local estimates of the coefficients specific to district *i* in a unifying framework, as given by Eq. (6).

6 $${y}_{i}={\beta }_{0}\left({u}_{i},{v}_{i}\right)+\sum_{j=1}^{k}{\beta }_{j}\left({u}_{i},{v}_{i}\right){x}_{ij}+{\varepsilon }_{i}$$

where ${\beta }_{0}\left({u}_{i},{v}_{i}\right)$ are estimated intercepts, ${\beta }_{j}\left({u}_{i},{v}_{i}\right)$ are GWR coefficients in district *i*, and $\left({u}_{i},{v}_{i}\right)$ are the latitude and longitude coordinates of the centroid of district *i*.

The underlying hypothesis in the model is that the geographical proximity of two observations will have an impact on how similar the explanatory variables are to the response variable or how near the explanatory variable coefficients are to one another. A kernel function is used in the GWR model to assign a decreasing weight with the distance for remote geographical locations relative to the location of interest. The shape of the kernel, whether fixed or adaptive, and bandwidth size are the key parameters that need to be determined. If the bandwidth size is too large, then a large proportion of the study area may be included, resulting in non-significance of local heterogeneity.

Since our study area consists of 40 districts, and the district level is the finest spatial scale whereby the COVID-19 and socio-demographic data are available, the adaptive Gaussian kernel with bandwidth size 12 is chosen for discovering the local heterogeneity in the GWR model implemented with GWmodel package [47]. This selection is partly attributed to the fact that a district in the study area has, on average, 11.3 second-order queen contiguity neighbors, besides balancing the bias-variance tradeoff. As Sarawak was placed under inter-district travel ban most of the time in 2021, extending the number of neighbors or bandwidth may not be feasible.

### Implementation of analysis

**Fig. 3:**
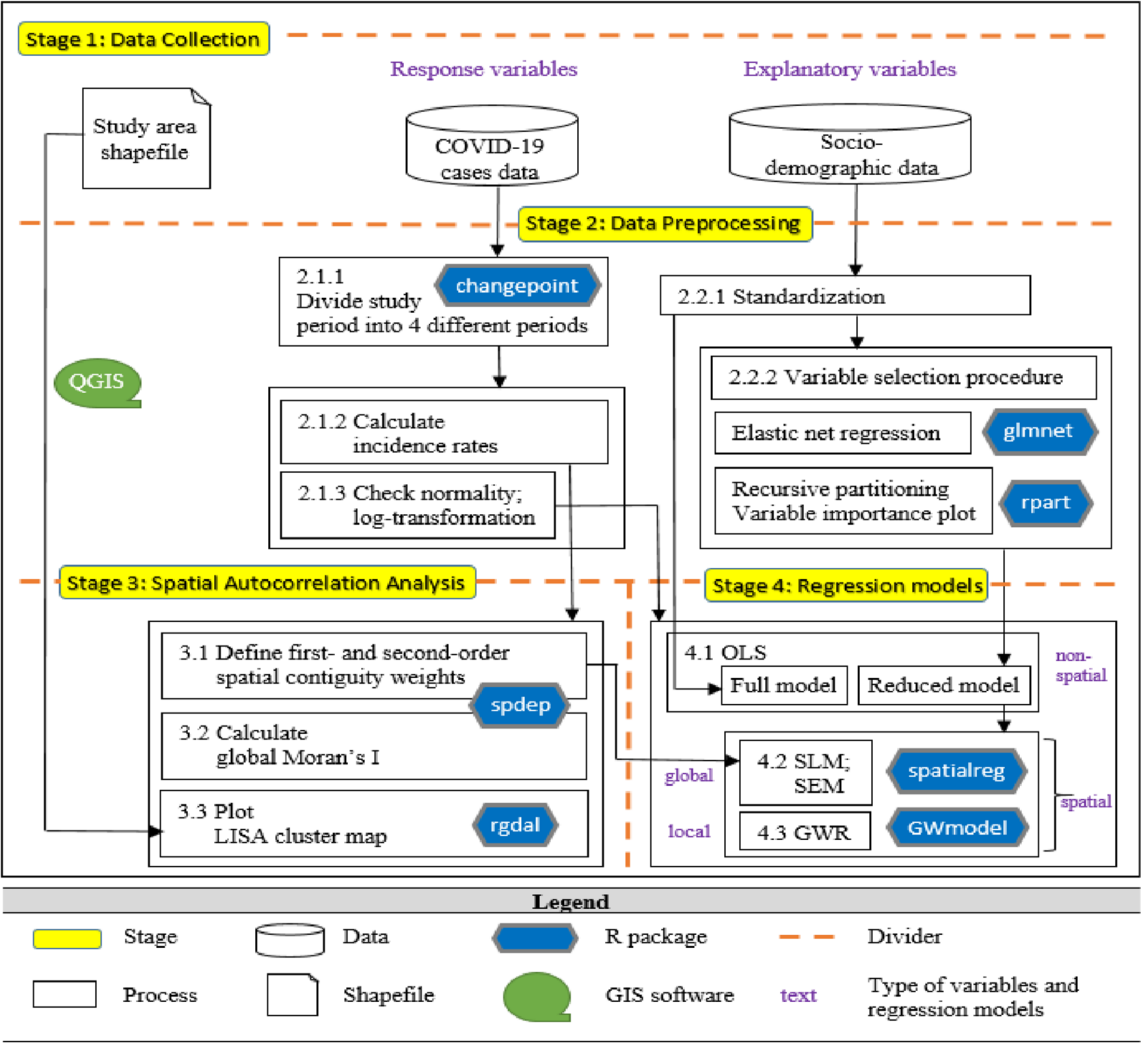
Flowchart for the summary of the methodology

The data preprocessing, variable selection, spatial autocorrelation and regression modelling in this study were all implemented in R. Figure 3 shows the flowchart for summarizing the methodology mentioned above. Apart from the assessment of different orders of spatial weights in capturing the spatiotemporal autocorrelation and detecting the high-risk districts, the analysis will also include the evaluation of the significance of coefficients and model fitting across different temporal periods.

## Results

### Change point analysis and normality checking for response variables

**Fig. 4:**
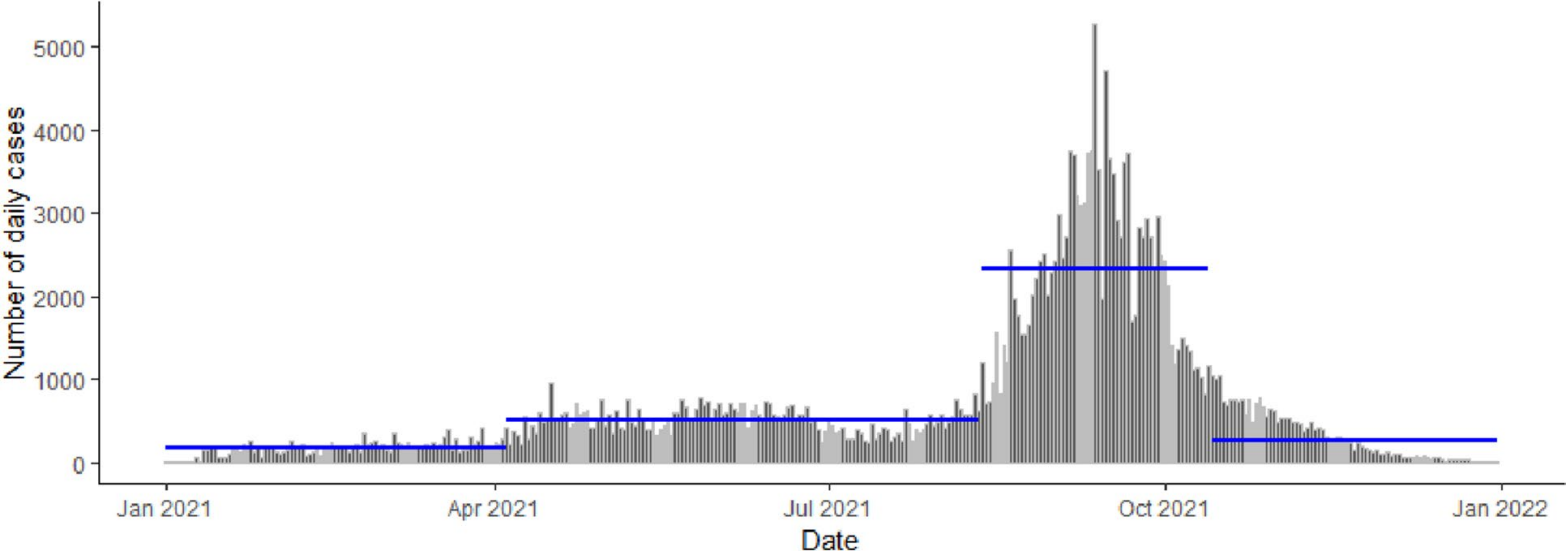
Abrupt changes in mean and variance for daily confirmed COVID-19 cases in Sarawak throughout 2021 occurred on 4 April, 12 August, and 14 October 2021. The blue horizontal line indicates the mean of daily cases in a particular period

The COVID-19 data for 2021 is divided into four periods by performing change point analysis. The abrupt changes in mean and variance occurred on 4 April, 12 August and 14 October 2021 (see Fig. 4). Sarawak recorded thousands of daily cases between August and October 2021. The subsequent analysis is conducted for the four designated periods within the year, whereby Table 2 displays their brief overview. This segmentation of periods is somehow not far from the global waves defined in [22] (see the last column of Table 2). Hence, the terms period and wave will be used interchangeably in this study.

**Table 2:** Summary of different waves during the COVID-19 pandemic in Sarawak throughout 2021

	**Period**	**Number of days**	**Average number of daily cases**	**Range of district-wise incidence rate**	**Global waves classified in **[22]
Year 2021	1 Jan – 31 Dec	365	688.10	[21.46, 255.99]	
Wave 1	1 Jan –3 Apr	93	172.07	[0.05, 33.73]	July 2020 to Feb 2021
Wave 2	4 Apr – 11 Aug	130	509.56	[1.46, 93.26]	Mar to June 2021
Wave 3	12 Aug – 13 Oct	63	2339.88	[7.06, 120.87]	July to Oct 2021
Wave 4	14 Oct – 31 Dec	79	272.15	[1.09, 24.72]	Nov 2021 to Mar 2022

**Fig. 5:**
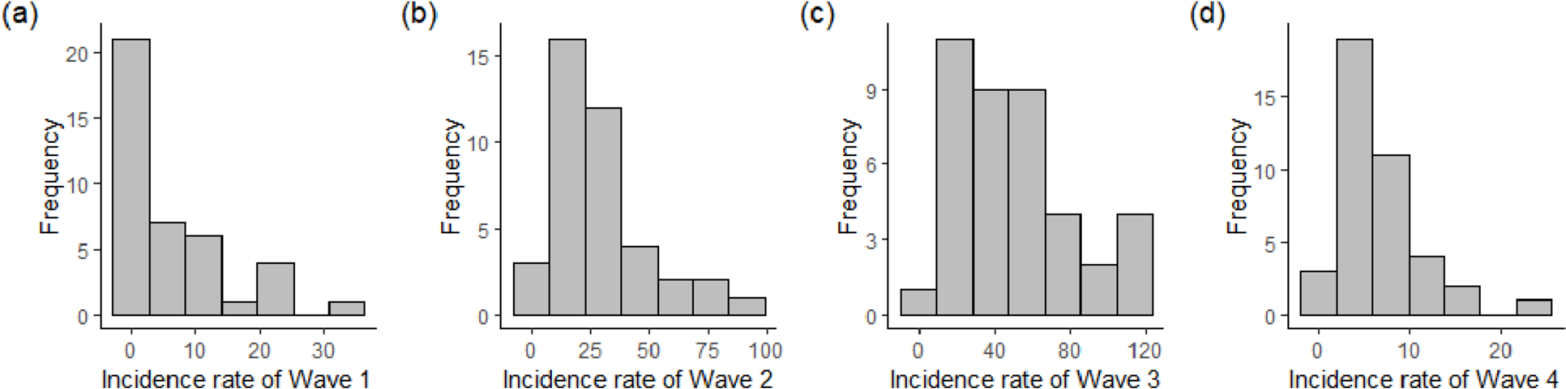
The histograms for incidence rates in different waves and their Shapiro–Wilk test *p*-value (**a**) 7.239 × 10^–7^, (**b**) 0.0007786, (**c**) 0.02905, and (**d**) 0.000212, respectively

**Fig. 6:**
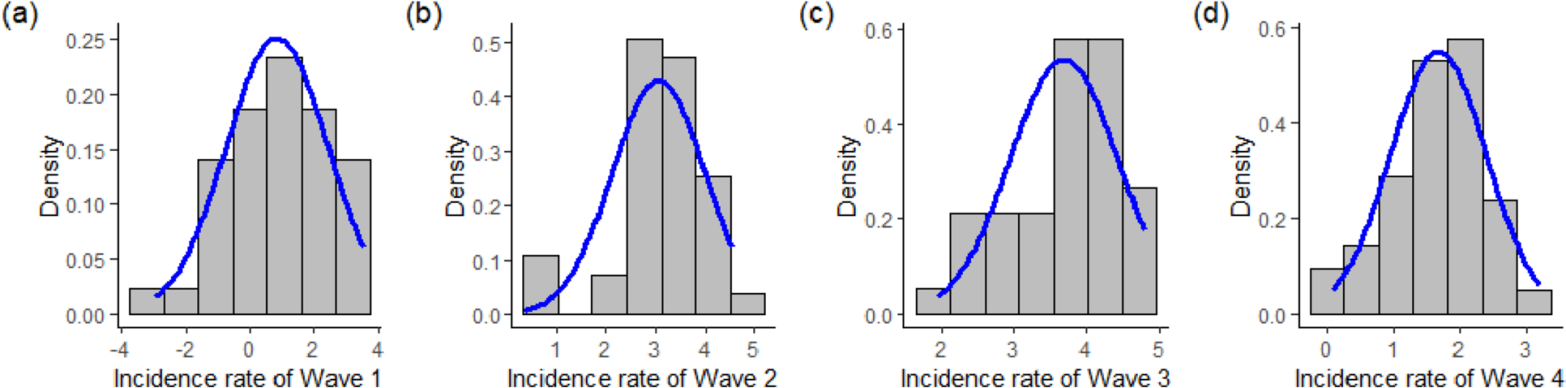
The histograms for log-transformed incidence rates in different waves and their Shapiro–Wilk test *p*-value (**a**) 0.3235, (**b**) 0.007643, (**c**) 0.05231, and (**d**) 0.869, respectively

All the incidence rates in four different waves are either skewed to the right or exponentially distributed, and their respective Shapiro–Wilk *p*-values are less than 0.05 (see Fig. 5). Hence, the district-wise incidence rates are log-transformed to obtain relatively less skewed but approximately normally distributed response variables in this study. All the incidence rates can be considered normally distributed, except for Wave 2, after the logarithmic transformation (see Fig. 6).

### Variable selection for explanatory variables

**Fig. 7:**
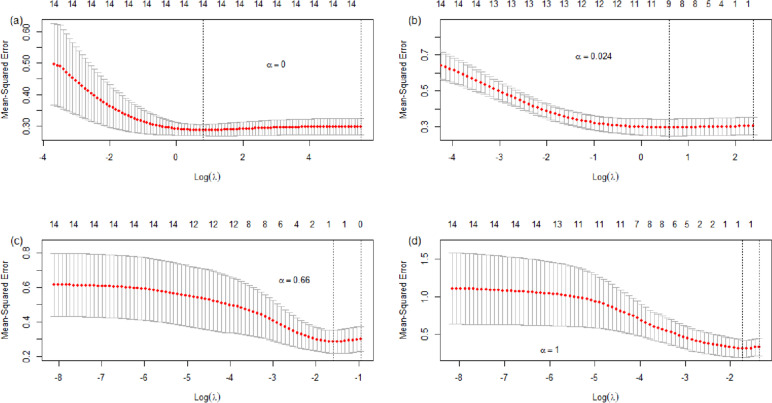
Mean squared error (MSE) versus log(λ) for specified α: (**a**) 0, (**b**) 0.024, (**c**) 0.66, and (**d**) 1

The best subset of explanatory variables is determined by both the regularization technique and recursive partitioning. The cross-validation curve (red dotted line) with evaluation metrics mean square error (MSE) for four different alpha (α) values are depicted in Fig. 7. The two vertical dotted lines indicate the parameter lambda (λ) that minimizes the cross-validation prediction error rate (CV-E) and CV-E within one standard error of the minimum in the graph. For instance, at α = 0 (see Fig. 7(a)), minimum CV-E occurs at log(λ) = 0.799 with 14 variables (see the top of the left vertical dotted line) for ridge regression. This high number of variables indicates the full model is probably the best if α = 0 is chosen.

Hence, another α is explored by gradually increasing its value to obtain the corresponding parameter λ that gives a significant reduction in the number of explanatory variables. To avoid overfitting as well as underfitting, the elastic net regression with α = 0.024 is chosen. It produces an optimal set of coefficients with nine explanatory variables, as indicated by the number 9 at the top of the left vertical line in Fig. 7(b). Larger α values such as 0.66 and 1 (see Figs. 7(c) and (d)) tend to leave out all the explanatory variables by remaining only the intercept.

**Fig. 8:**
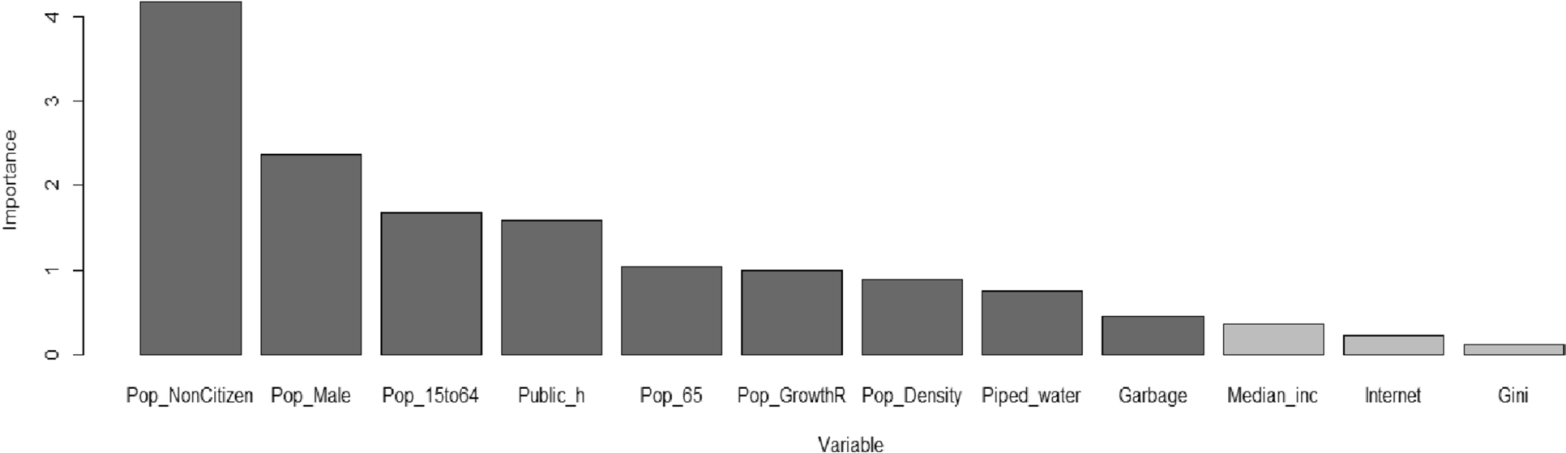
Variable importance plot for single regression tree

We fit a regression tree using a recursive partitioning approach and provide a variable importance plot in Fig. 8 to identify which nine factors, among all 14 explanatory variables, to be included in subsequent modelling. The variable importance plot gives the list of the most significant variables in descending order. The first nine explanatory variables ranging from Pop_NonCitizen to Garbage will be selected as they have higher predictive power for incidence rate in 2021 than the rest of the five explanatory variables.

### Linear non-spatial regression model

Table 3 shows the comparison between the full and reduced linear ordinary least square (OLS) regression models. Since the explanatory variables are standardized and the response variable is logarithmically transformed, the estimated coefficients should be interpreted as follows: Keeping all the other explanatory variables constant, on average, a one standard deviation change in the explanatory variable concerned is associated with a change in the exponential of incidence rate. Three explanatory variables (i.e., Pop_NonCitizen, Public_h and Garbage) are significant in the reduced linear regression (with nine explanatory variables) compared to only one variable (Pop_NonCitizen) in the full model (with 14 explanatory variables). The multiple R-squared is expected to be higher in the full model with a higher number of explanatory variables. However, as its adjusted R-squared is lower, the model accuracy of reduced linear regression is better than its corresponding full model, although the reduced model can only explain 17% of the variance in incidence rate 2021.

**Table 3:** Regression coefficient estimates and statistics for full and reduced linear non-spatial regression

Variable	Coefficients		Collinearity statistics
	**Full model**	**Reduced model**	**VIF (reduced model)**
(Intercept)	4.403 ***	4.403 ***	
Pop_GrowthR	-0.013	-0.046	1.314
Pop_NonCitizen	-0.304 **	-0.267 **	2.468
Pop_Male	0.129	0.096	2.591
Pop_15to64	-0.258	-0.198	3.230
Pop_65	0.004	0.031	2.232
Pop_Density	0.119	0.126	2.213
Household_size	-0.058		
Median_inc	0.102		
Gini	-0.008		
Poverty	-0.101		
Internet	-0.257		
Piped_water	0.246	0.028	2.534
Public_h	-0.267	-0.311 **	3.456
Garbage	0.128	0.225 *	2.559
**Regression statistics**			
Multiple R-squared	0.428	0.362	
Adjusted R-squared	0.107	0.171	

^*^p-value < 0.1

^**^p-value < 0.05

^***^p-value < 0.01

The multi-collinearity in the reduced model is measured by finding variance inflation factor (VIF) using car (i.e., Companion to Applied Regression) package [48] in R. If the VIF is less than 3, then the correlation is low among variables under ideal conditions. Typically, only variables with VIF less than 5 will be included in the model. From the last column of VIF in Table 3, the nine explanatory variables selected in the variable selection procedure could be considered free of multi-collinearity issues.

**Fig. 9:**
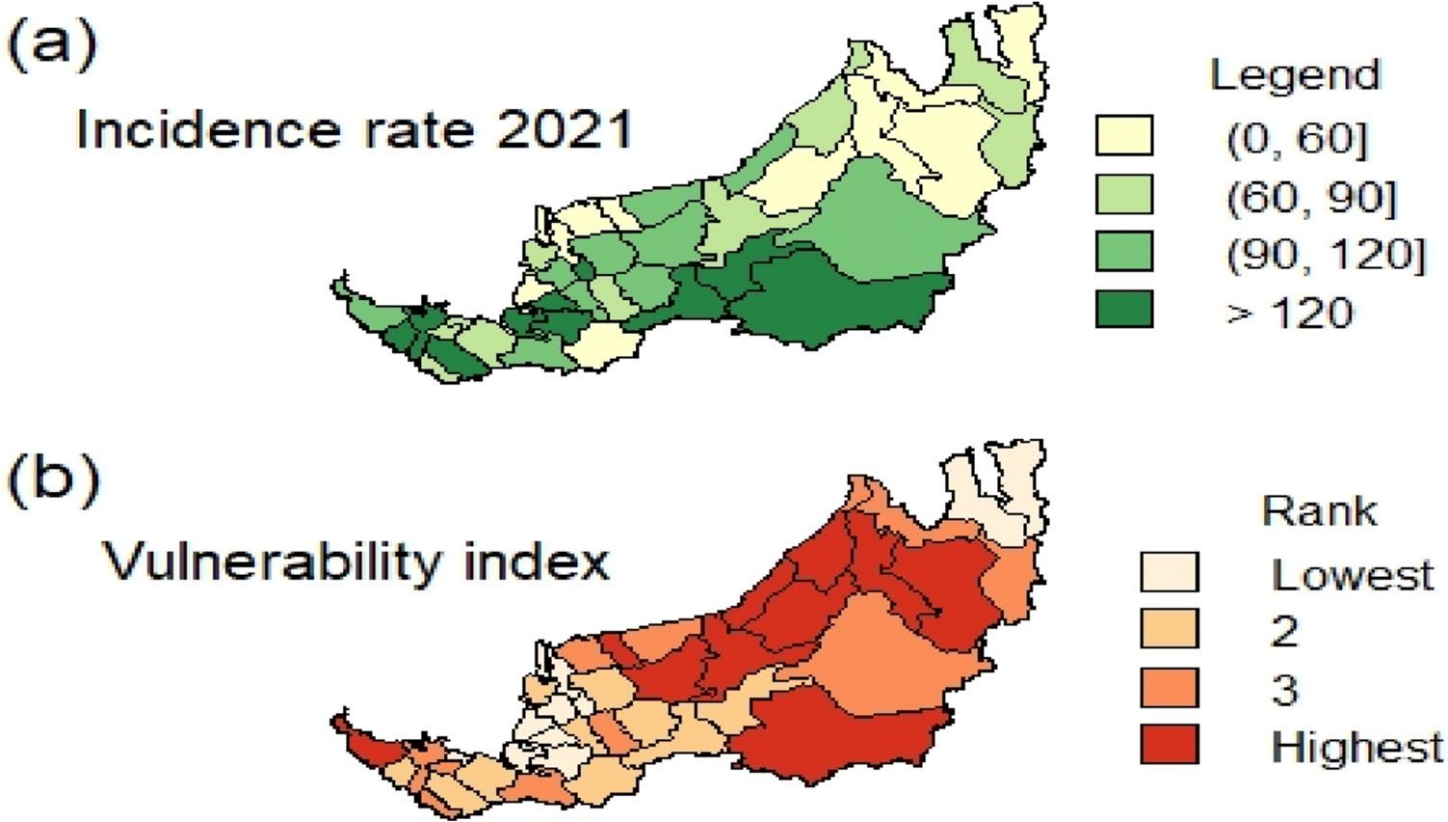
**a** Incidence rate in 2021 for each district in Sarawak. **b** Vulnerability index map for all districts in Sarawak

Figure 9 depicts the spatial variation of 40 districts’ incidence rate in 2021 and vulnerability index. The vulnerability index normalization is carried out before an equal weighting scheme is implemented for all the nine selected explanatory variables. This allows us to determine the most vulnerable districts based on the selected explanatory variables. In Figs. 9(a) and (b), the highest incidence rate districts are mostly located in the southern and inner parts of Sarawak, whereas a considerable number of coastal districts in the middle to northern parts are most vulnerable. Three districts, namely, Kabong, Daro and Lawas, can be classified as least vulnerable and lowest incidence rates throughout 2021. Bukit Mabong is found to be the most vulnerable and recorded the highest incidence rate. These reflect that the selected explanatory variables and their associated vulnerability map may be useful for epidemic tracing. However, a more comprehensive vulnerability index with more sophisticated weighting schemes and additional variables could be further refined to provide valuable insights into the potential relationship between vulnerability and disease incidence in Sarawak.

### Global spatial autocorrelation analysis

Table 4 shows Moran’s I statistic and its *p*-value for incidence rates in different temporal periods using two different orders of queen contiguity spatial weights. All Moran’s I statistics calculated in Table 4 indicate that its incidence rate tends to cluster (i.e., positive spatial autocorrelation), and they are statistically significant with *p*-values < 0.05. However, the incidence rate for Wave 4 under the second-order contiguity spatial weight shows no significant spatially autocorrelated, with *p*-value = 0.229 > 0.05. This may imply that a small number of neighboring districts is already sufficient to capture its spatial autocorrelation in Wave 4, which has the smallest range of district-wise incidence rates (see Table 2). Overall, the findings in Table 4 suggest that the incidence rates among districts that are geographically adjacent or near are similarly high (resp. low) throughout 2021. This spatial clustering feature is strongest in Wave 2 based on first-order queen contiguity, but occurs in Wave 3 if second-order contiguity is used.

**Table 4:** Global spatial autocorrelation analysis using first- and second-order queen contiguity spatial weights

Queen contiguity		**Incidence rate**				
		2021	Wave 1	Wave 2	Wave 3	Wave 4
First-order	Moran’s I statistic	0.213	0.214	0.373	0.329	0.277
	*p*-value	0.022	0.021	1.288 × 10^–4^	6.676 × 10^–4^	0.003
Second-order	Moran’s I statistic	0.126	0.105	0.130	0.362	0.044
	*p*-value	0.009	0.026	0.008	4.016 × 10^–11^	0.229

### Local spatial autocorrelation analysis

**Fig. 10:**
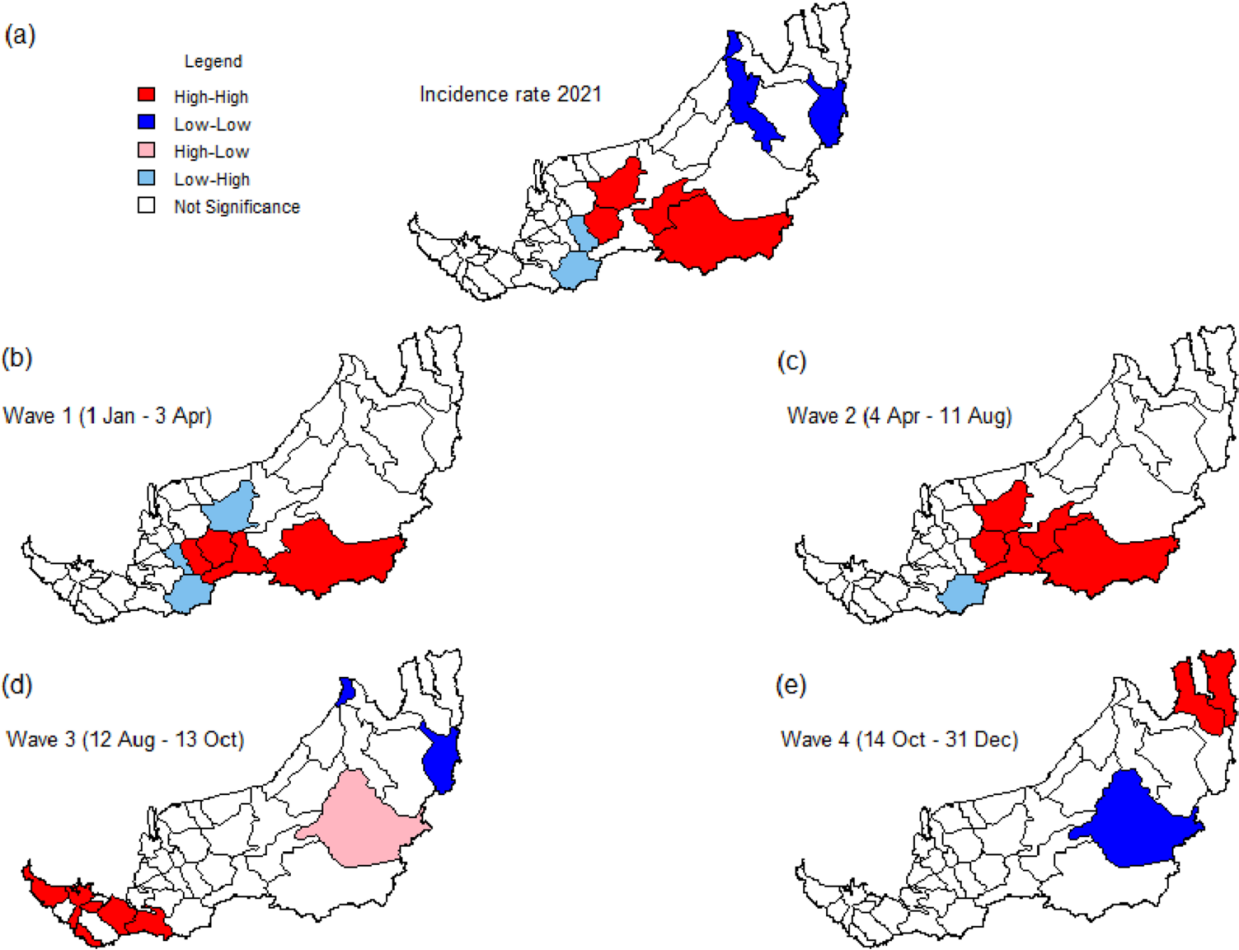
Local indicators of spatial association (LISA) cluster map for incidence rates in different periods based on first-order queen contiguity spatial weights

**Fig. 11:**
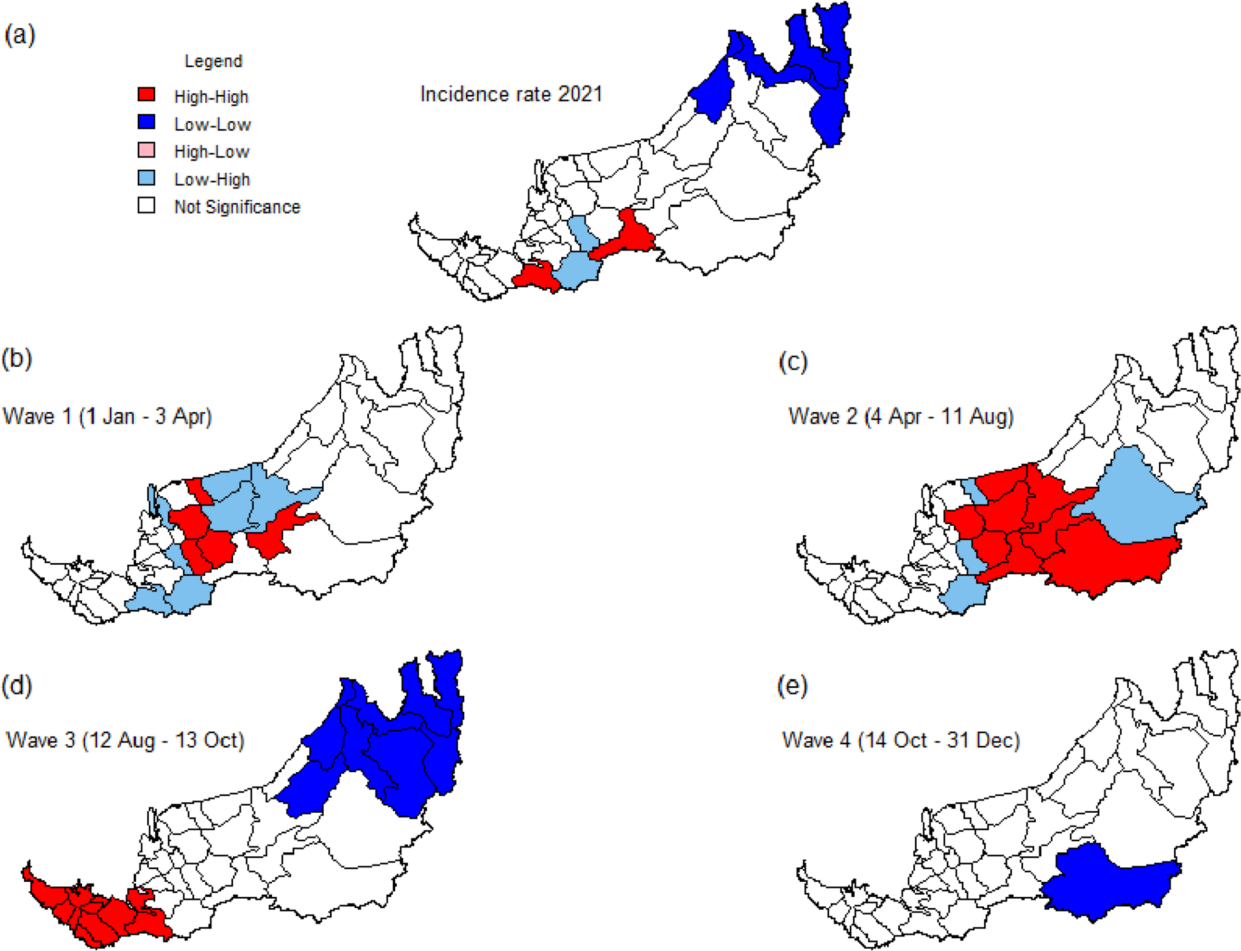
Local indicators of spatial association (LISA) cluster map for incidence rates in different periods based on second-order queen contiguity spatial weights

The local indicators of spatial association (LISA) cluster map was obtained by using the first- and second-order queen contiguity spatial weights as shown in Figs. 10 and 11, respectively. The strongly (red and blue) colored districts contribute significantly to the positive local spatial autocorrelation, whereas the paler colors (pink and light blue) represent the negative local spatial autocorrelation.

We first look at the red-colored districts in Fig. 10, which correspond to a significant spatial cluster of districts with a high incidence rate. These districts (Julau, Kanowit, Song, Bukit Mabong, Sibu, and Kapit) are mostly concentrated in the central region of Sarawak during the first and second waves but shift to the southern part of Sarawak (including district Lundu, Kuching, Samarahan, Tebedu, Simunjan, and Sri Aman) in the third wave (see Figs. 10(b)-(d)). The spatial dependence structure via first-order spatial weights only leads to very few low-low clusters, mainly occurring in the districts in the northern region. These include Miri and Beluru if the entire year 2021 is considered and Miri and Belaga in Waves 3 and 4, respectively. Conversely, Belaga is the only district found to be potentially spillover disease to its neighboring districts during Wave 3 (see pink colored district in Fig. 10(d)).

If the second-order queen contiguity spatial weight is used, the existence of such spatial clusters is more prominent. Five districts (Dalat, Sibu, Kanowit, Julau, and Kapit) and eight districts (Mukah, Sibu, Selangau, Tatau, Kanowit, Song, Kapit, and Bukit Mabong) in the central region can be categorized as high-high cluster in Waves 1 and 2, respectively. Additionally, a sizable number of low–high spatial outliers emerged in the first and second waves (see light blue colored districts in Figs. 11(b) and (c)). In Wave 3, spatially homogeneous high-high clusters appeared in all 10 districts in southern Sarawak, in contrast to a low-low cluster that emerged in the northern part (Fig. 11(d)). This obvious gradient effect might suggest that the disease risk is highly spatially structured in that period. In Wave 4, only one district, namely Bukit Mabong is found to be significantly low-low (Fig. 11(e)).

Although Moran’s I statistics of second-order spatial weight are mostly lower than its first-order counterpart (see Table 4), a more visible pattern of spatial clusters and outliers is observed. This suggests that second-order contiguity weight is more suitable to detect high-risk spatial–temporal districts in this study. The results are more consistent with the co-evolution of COVID-19 cases throughout Sarawak, if the analysis is based on the second-order queen contiguity.

In brief, several districts in the central part (resp. northern region) of Sarawak have high (resp. low) incidence rates and have neighboring districts that also have high (resp. low) incidence rates throughout 2021 based on both orders of queen contiguity spatial weights. The temporal evolution of districts with significant spatial clusters across four different periods indicates COVID-19 hit the central region of Sarawak seriously in the first 8 months of 2021, followed by the southern region of Sarawak in Wave 3 in the following 2 months.

### Global spatial regression (SLM and SEM)

Table 5 shows the coefficient estimates and statistical test values for the SLM. The coefficients estimated are stable between two different orders of spatial weights. However, apart from varying significance, the coefficients do not necessarily show the same sign across five different temporal periods. This contrasting result is particularly obvious for variables Pop_NonCitizen, Pop_Male, Pop_65 and Piped_water. While holding other variables in the SLM constant and focusing only on the statistically significant variables, we find that as the Pop_NonCitizen (resp. Pop_15to64; Public_h) increases, the district-wise incidence rates tend to decrease in the entire 2021 and Wave 3 (resp. Waves 1 and 4; entire 2021, Waves 1 and 2). Conversely, the variable Garbage (resp. Piped_water) has a positive effect on incidence rates in the entire 2021, Waves 1, 2, and 4 (resp. Wave 4).

**Table 5:** Coefficient estimates and statistical test values for the spatial lag model (SLM) based on first- and second-order queen contiguity spatial weights

	Spatial weights	2021	Wave 1	Wave 2	Wave 3	Wave 4
Intercept	First-order	3.550 ***	0.563 **	1.674 ***	2.257 ***	1.159 ***
	Second-order	3.163 ***	0.692 **	1.903 **	1.239 **	2.116 ***
Pop_GrowthR	First-order	-0.056	-0.288	0.001	-0.151	-0.156
	Second-order	-0.058	-0.275	-0.018	-0.152	-0.133
Pop_NonCitizen	First-order	-0.244 **	0.453	-0.157	-0.261 *	-0.035
	Second-order	-0.258 **	0.484 *	-0.171	-0.284 **	-0.046
Pop_Male	First-order	0.095	-0.006	0.050	0.091	0.222
	Second-order	0.102	-0.000	0.049	0.119	0.165
Pop_15to64	First-order	-0.201	-0.829 ***	-0.054	-0.097	-0.465 ***
	Second-order	-0.510 *	-0.817 **	-0.076	-0.126	-0.417 ***
Pop_65	First-order	-0.004	0.283	0.139	-0.038	-0.085
	Second-order	-0.003	0.366	0.179	-0.060	-0.057
Pop_Density	First-order	0.101	0.351	0.053	0.134	0.004
	Second-order	0.101	0.367	0.098	0.077	-0.063
Piped_water	First-order	0.044	-0.016	-0.237	0.178	0.321 **
	Second-order	0.035	-0.082	-0.273	0.172	0.338 **
Public_h	First-order	-0.293 **	-1.297 ***	-0.482 **	-0.112	-0.161
	Second-order	-0.309 **	-1.326 ***	-0.564 ***	-0.150	-0.077
Garbage	First-order	0.235 **	1.399 ***	0.572 ***	0.047	0.335 **
	Second-order	0.239 **	1.414 ***	0.595 ***	0.073	0.265 *
Rho	First-order	0.190	0.264	0.440	0.380	0.300
	Second-order	0.279	0.150	0.367	0.659	-0.270
LR test value	First-order	0.668	1.593	5.196 **	2.781 *	2.344
	Second-order	0.837	0.248	1.708	6.990 ***	0.375
Log-likelihood	First-order	-22.731	-60.933	-41.926	-36.342	-31.406
	Second-order	-22.646	-61.605	-43.670	-34.238	-32.391
AIC	First-order	69.462	145.87	107.85	96.685	86.814
	Second-order	69.293	147.21	111.34	92.477	88.781
(AIC for lm)		(68.13)	(145.46)	(111.05)	(97.467)	(87.158)
RMSE	First-order	0.425	1.100	0.672	0.589	0.524
	Second-order	0.424	1.127	0.715	0.552	0.542
LM test for residual autocorrelation	First-order	3.575 *	0.001	0.000	0.382	0.003
	Second-order	0.841	4.248 **	1.681	2.215	1.708

^*^p-value < 0.1

^**^p-value < 0.05

^***^p-value < 0.01

By comparing their log-likelihood, Akaike information criterion (AIC) and root mean square error (RMSE) values for different temporal periods, our results consistently show a better model fit to the incidence rates for the entire year of 2021 than for the respective four waves. For the comparison between two different spatial weight definitions, the first-order spatial weights outperform the second-order during Waves 1, 2 and 4. The incidence rate in a district is strongly influenced by its neighboring district rates in the second wave based on the first-order spatial weights. However, the strongest influence occurs in Wave 3 based on the second-order spatial weights, in which not only the AIC of SLM (92.477) is lower compared to its corresponding non-spatial linear model (97.467), but also their likelihood ratio (LR) tests return significant results (i.e., the addition of the spatial lag is an improvement to the model). Its rho parameter value is also the highest (0.659).

The Lagrange multiplier (LM) test for residual autocorrelation is significant only for the incidence rate of 2021 (with first-order contiguity) and the incidence rate in Wave 1 (with second-order contiguity). The null hypothesis of randomly distributed residuals can therefore be rejected in these two scenarios of SLM. This suggests that the residuals could be spatially structured in the other waves or that a different spatial weight definition is used. Hence, we proceed by presenting the results of SEM.

Similar to SLM, the coefficients estimated in SEM are stable between two different orders of spatial weights (see Table 6). However, the contrasting effect (i.e., the coefficients with different signs) across different waves can be found for all variables except Pop_15to64 and Garbage. The statistically significant variables in SEM appear to be quite identical to those detected in SLM, with additional significant variables Pop_GrowthR and Piped_water in Wave 3.

**Table 6:** Coefficient estimates and statistical test values for the spatial error model (SEM) based on first- and second-order queen contiguity spatial weights

	Spatial weights	2021	Wave 1	Wave 2	Wave 3	Wave 4
Intercept	First-order	4.433 ***	0.809 ***	3.007 ***	3.508 ***	1.671 ***
	Second-order	4.398 ***	0.886 ***	3.032 ***	3.577 ***	1.662 ***
Pop_GrowthR	First-order	0.056	-0.252	0.004	-0.214 **	-0.149
	Second-order	-0.051	-0.251	-0.018	-0.170 *	-0.177 *
Pop_NonCitizen	First-order	-0.320 ***	0.464	-0.138	-0.174	-0.042
	Second-order	-0.262 **	0.507 *	-0.140	-0.273 **	0.005
Pop_Male	First-order	0.111	-0.046	0.033	0.101	0.205
	Second-order	0.097	0.175	0.045	0.123	0.125
Pop_15to64	First-order	-0.266 **	-0.765 **	-0.050	-0.084	-0.431 ***
	Second-order	-0.200	-1.050 ***	-0.080	-0.114	-0.412 **
Pop_65	First-order	0.069	0.291	0.170	-0.228	-0.038
	Second-order	-0.025	0.519 **	0.181	-0.060	-0.121
Pop_Density	First-order	0.198 **	0.270	0.008	-0.146	0.037
	Second-order	0.114	0.171	0.107	-0.028	-0.099
Piped_water	First-order	-0.030	-0.030	-0.271	0.244 *	0.314 **
	Second-order	0.039	-0.167	-0.291	0.261 *	0.331 ***
Public_h	First-order	-0.483 ***	-1.214 ***	-0.394 **	0.080	-0.144
	Second-order	-0.309 **	-1.519 ***	-0.551 ***	-0.146	-0.025
Garbage	First-order	0.196 *	1.389 ***	0.594 ***	0.045	0.286 **
	Second-order	0.222 **	1.871 ***	0.583 ***	0.062	0.274 **
Lambda	First-order	-0.845	0.264	0.486	0.736	0.309
	Second-order	0.127	-1.105	0.310	0.774	-0.994
LR test value	First-order	1.675	0.962	4.722 **	7.007 ***	1.253
	Second-order	0.061	1.274	0.755	7.633 ***	1.966
Log likelihood	First-order	-22.227	-61.249	-42.163	-34.229	-31.952
	Second-order	-23.034	-61.093	-44.147	-33.916	-33.595
AIC	First-order	68.455	146.5	108.33	92.459	87.905
	Second-order	70.069	146.19	112.29	91.833	87.191
(AIC for lm)		(68.13)	(145.46)	(111.05)	(97.467)	(87.158)
RMSE	First-order	0.390	1.109	0.672	0.521	0.531
	Second-order	0.430	1.067	0.725	0.538	0.514

^*^p-value < 0.1

^**^p-value < 0.05

^***^p-value < 0.01

It is found that Wave 2 (with first-order spatial weights) and Wave 3 (with both orders of spatial weights) not only have significant spatial error autocorrelation parameters but also have lower AIC values than the corresponding non-spatial linear models. Other spatially-structured predictors might need to be added to the model to make it better, especially for Waves 2 and 3.

### Local spatial regression model (GWR)

Table 7 presents the range of coefficient estimates and statistical test values for the adaptive Gaussian kernel GWR with a bandwidth of 12. All GWR coefficients for explanatory variables estimate exhibit locally different signs and are significant (except Pop_Male and Pop_15to64) in at least one of the temporal periods. The findings imply that using local spatial regression such as GWR is essential to comprehend the spatial variation in the local relationships between district-wise incidence rates and socio-demographical indicators.

**Table 7:** Range of coefficient estimates and statistical test values for geographically weight regression (GWR) with bandwidth = 12

	**2021**	**Wave 1**	**Wave 2**	**Wave 3**	**Wave 4**
Intercept	[4.37, 4.46]	[0.54, 1.40]	[2.90, 3.30]	[3.59, 3.89]	[1.60, 1.95]
Pop_GrowthR	[-0.20, -0.01]	[-0.75, -0.21]	[-0.10, 0.11]	[-0.26, -0.09]	[-0.44, 0.00]**
Pop_NonCitizen	[-0.85, -0.23]**	[-0.99, 0.71]	[-0.83, -0.16]	[-1.25, 0.28]***	[-0.07, 0.41]
Pop_Male	[-0.05, 0.39]	[-0.31, 0.27]	[-0.22, 0.19]	[-0.07, 0.65]	[-0.10, 0.26]
Pop_15to64	[-0.29, -0.07]	[-1.37, -0.63]	[-0.21, 0.11]	[-0.22, 0.10]	[-0.46, -0.23]
Pop_65	[-0.26, 0.13]***	[-0.58, 0.86]***	[-0.54, 0.49]***	[-0.24, 0.11]	[-0.30, 0.22]**
Pop_Density	[-0.03, 0.24]	[-0.26, 1.25]***	[-0.57, 0.49]***	[ 0.09, 0.32]	[-0.19, 0.20]
Piped_water	[-0.46, 0.20]***	[-0.80, 0.19]	[-0.73, -0.15]	[-0.49, 0.43]***	[ 0.11, 0.40]
Public_h	[-0.37, -0.12]	[-1.73, -1.00]	[-1.00, -0.28]*	[-0.16, 0.16]	[-0.42, 0.23]**
Garbage	[0.10, 0.57]***	[ 1.19, 2.34]**	[0.61, 0.94]	[-0.16, 0.32]*	[0.08, 0.33]
Adjusted R2	0.313	0.422	0.289	0.169	0.29
(Adj R2 for lm)	(0.171)	(0.326)	(0.166)	(0.070)	(0.242)
AIC	32.021	110.695	76.063	64.371	55.642
(AIC for lm)	(68.130)	(145.460)	(111.050)	(97.467)	(87.158)
F4 test *p*-value	0.036 *	0.042 *	0.041 *	0.051	0.060

^*^p-value < 0.05

^**^p-value < 0.01

^***^p-value < 0.001

**Fig. 12:**
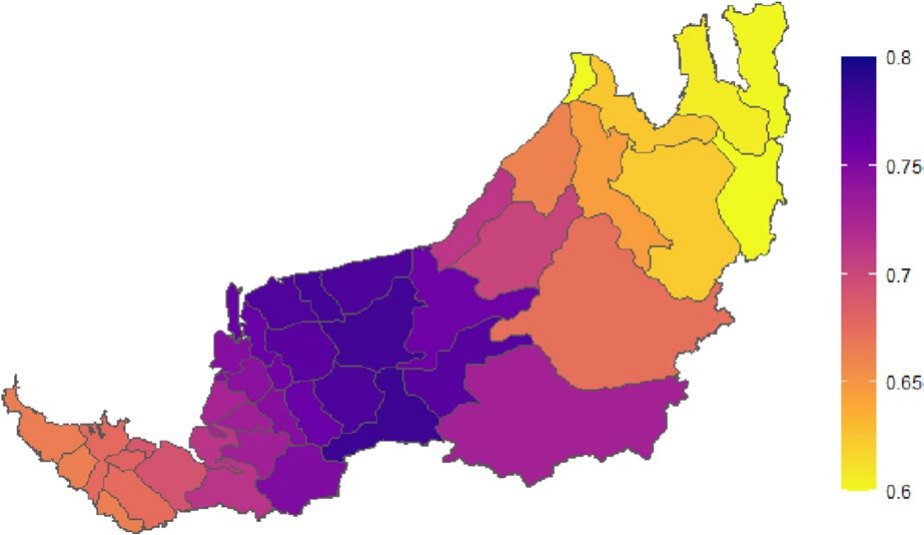
R-squared values of the GWR model for incidence rate 2021

The GWR’s adjusted R-squared in different temporal periods is higher than its corresponding non-spatial linear model. It shows that the GWR improves the model’s accuracy despite the highest percentage of variance in the incidence rates explained by the selected nine explanatory variables, which is 42% in Wave 1. The adjusted R-squared is the lowest in Wave 3, with only approximately 17%, possibly due to Wave 3 having the largest range and greater spatial non-stationary of district-wise incidence rates (see Table 2 and Fig. 11(d)). AIC for GWR in all temporal periods are lower than their corresponding non-spatial regression model. Moreover, the F4 test *p*-values are significant for the entire 2021, Waves 1 and 2 indicating there are significant improvements in the residual sum of squares in the local model over the global OLS model. Meanwhile, as far as the entire year of 2021 is concerned, the local spatial regression model fits better for the districts in the central region than those in the southern or northern regions of Sarawak (see Fig. 12).

**Fig. 13:**
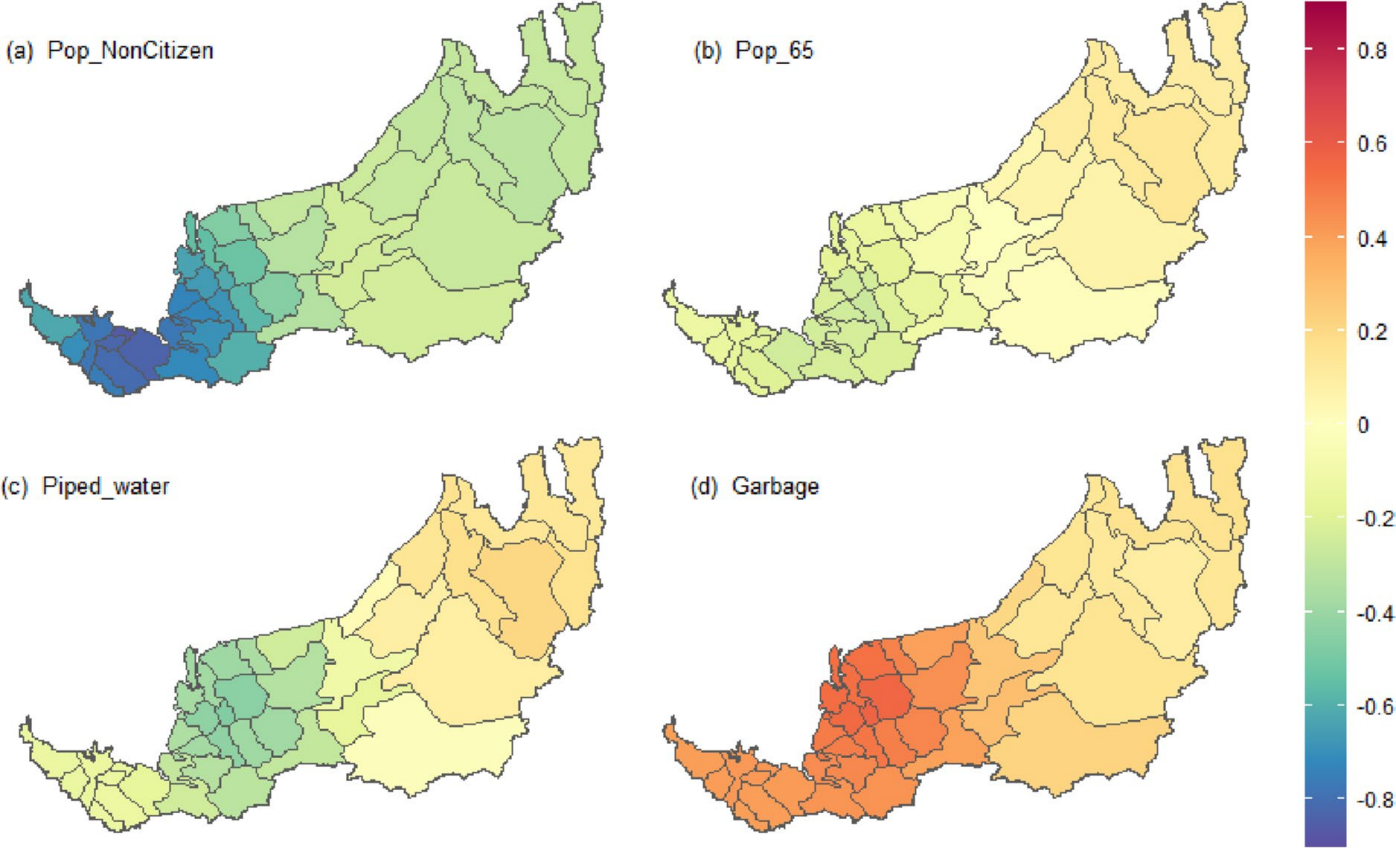
GWR coefficient surfaces for highlighting the relationship between four selected explanatory variables and the incidence rate in 2021

GWR has a surface of parameter estimates, rather than one single estimate for each explanatory variable. Hence, Fig. 13 highlights the strength and direction of the relationship between four selected explanatory variables and the incidence rate in 2021. All the relationships between the selected explanatory variables and the incidence rate in 2021 in Fig. 13 have clear geographical variation. It indicates that the locally changing influence of the explanatory variables on the incidence rate in some districts might be much stronger than in others.

Although the global regression coefficient estimate for variables Pop_65 and Piped_water are positive (0.031 and 0.028, respectively; see the column of the reduced model in Table 3), its local regression coefficient estimates are ranging from -0.26 to 0.13 and from-0.46 to 0.20, respectively (see the column of 2021 in Table 7). The evidence for relationship non-stationarity could be visualized through the coefficient surface plots as given in Figs. 13(b) and (c). When variables Pop_65 and/or Piped_water increase, the incidence rates for districts in the northern part of Sarawak are expected to increase, but for districts in the central and southern parts of Sarawak, their incidence rates may decrease. Notably, the parameter estimates for these two variables are close to 0 in certain districts, which indicates that the changes in Pop_65 and Piped_water do not influence changes in the districts’ incidence rate. Conversely, the local regression coefficients for Pop_NonCitizen are more negative in the southern part of Sarawak, whereas lower regression coefficients are found for variable Garbage in the northern part of Sarawak.

## Discussion

This study explores district-level spatial heterogeneity and the association between COVID-19 incidence rates and socio-demographic factors in Sarawak. The analysis was conducted using spatial autocorrelation and regression models, which include the global non-spatial linear, global spatial lag and spatial error, and local geographically weighted regression. The geographical proximity is quantified using either first- or second-order spatial weights in global spatial models. The incidence rates among districts that are close and connected are closer than districts that are not geographically adjacent and relatively far. Although the observations are robust across different settings throughout 40 districts in Sarawak and four different temporal periods in 2021, they nevertheless exhibit different or even contrasting results spatially and temporally.

### Spatial autocorrelation analysis

Global spatial autocorrelations of Sarawak district-wise incidence rates are most significant in Waves 2 and 3, based on first- and second-order contiguity, respectively. Conversely, from the LISA cluster maps in Figs. 10 and 11, the earliest spatiotemporal clustering of high district-wise incidence rates mainly occurs in central Sarawak, for the first 8 months of 2021. It should be acknowledged that a longhouse infection cluster, known as Pasai cluster, which was initially detected in Sibu and Mukah districts on 9 January 2021 and officially declared to have ended on 13 April 2021 by SDMC [49], emerged after a social gathering following a funeral event.

Behavioral epidemiology is increasingly seen as crucial to understanding infectious disease control. During the outbreak of Ebola hemorrhagic fever in Uganda, socio-cultural behaviors such as burial practices were seen as an important amplification factor for its spread [50]. Within the local customs in Sarawak, the acts of gathering during mourning are a sign of respect to the dead and the grieving family, and it may last a few days, until the body is buried. Effective preventive measures that are not socially or religiously acceptable are likely to face resistance and lose their effectiveness. This could be fueled by the lack of confidence between the health professionals and the affected community, where the disease is spreading [51]. A better understanding of disease transmission and having a higher risk perception, could have altered the people’s behavior, whereby paying the last respect to the dead may be done without physically congregating, but utilizing alternative virtual platforms.

Despite the end of the Pasai cluster in April, it became the epicenter of numerous other infection sub-clusters spanning nearby rural districts in Wave 2. Non-adherence to the preventive measures outlined by the State Government was seen as one of the reasons for the spread. Rural residents have been found to perceive a low risk of infection, or getting the COVID-19 disease despite having high levels of awareness about the COVID-19 virus and its transmission [52]. Rural dwellers were mainly concerned with issues related to the impact of the pandemic, such as pay cuts and loss of jobs. Moreover, limited internet coverage leads to the underuse of online media, making traditional news media such as radio and television remain as relevant as it was and the preferred choice among the elder generation in rural places. Traditional media, despite its relevancy, is more structured with evenly paced information, in contrast to online social media, which features continuous, unrestricted, and unfiltered information.

Besides non-adherence to preventive measures by the State Government, another factor that possibly contributed to the rapid spread of the infection cluster is the infectiousness of the new variant, coupled with the low level of population immunity. COVID-19 vaccination was only available to the mass public in Sarawak beginning in April 2021. The Pasai cluster was associated with the B.1.466.2 variant virus, which shares a mutation with the B.1.1.7 variant found in the United Kingdom and has a higher transmissibility. The B.1.466.2 variant was the predominant variant in the Pasai cluster, and was only limited geographically to Sarawak owing to the rigid restriction of interstate travel imposed after the onset of the third wave in Malaysia in 2020 [53]. The predominance of the strain in Sarawak coincides with and is similar to the circulating predominant strain in Indonesia from March to May 2021 [54]. It was suggested that the variant that predominates the Pasai cluster could have originated locally [55], further facilitating the rapidity of the virus transmission in a population where immunity is none [56].

Besides the B.1.466.2 variant that was predominate the infection clusters that emerged from central Sarawak in early 2021, the emergence of new variants of concern in a population where vaccination has just begun is a cause of concern since new variants are always associated with increased transmissibility, better immune evasion and higher virulence. The third wave in Sarawak has been attributed to the emergence of the Delta variant, which was first detected in Sarawak samples in June 2021. By August 2021, the Delta variant was predominantly isolated in Sarawak’s patient samples, coinciding with the ascending slope of the epidemiological curve of the third wave. The Delta strain is twice transmissible as the original virus, can produce an increased viral load in patients, replicates rapidly in the human host and displays a shorter incubation period [57]. These features could have explained the suspicion of the increased number of infections among those who have completed vaccination in the third wave [58] that mainly occurred in the southern region of Sarawak (see Fig. 11(d)), spreading up to the rural dominant central and northern part of the state. Hence, in the continuum of the spread, Limbang and Lawas showed the high-high spatial association in Wave 4 (Fig. 10(e)). Limbang and Lawas recorded clusters from prison inmates and among the longhouse dwellers, whereas generally Sarawak was in the decline in the trend of new COVID-19 cases.

### Spatial regression models

Similar to the global autocorrelation pattern, the Sarawak district-wise incidence rate is most strongly influenced by its neighboring district rates in Waves 2 and 3, based on first- and second-order contiguity weights in global spatial regression models, respectively. Conversely, the local spatial regression model improves the model accuracy for estimating incidence rates, especially in Wave 1 when compared to the OLS model.

Among all the nine selected explanatory variables, the garbage (i.e., percentage of households with garbage collection facilities within 100 m away from living quarters) appears to have a significant positive relationship with district-wise COVID-19 incidence rates for the majority of waves in 2021. This finding is aligned with the only study [59] that includes this factor as a social vulnerability indicator (as mentioned in the review paper [37]). Despite being rare in literature, garbage collection service reflects the population’s living conditions. It is an important public health infrastructure and an integral part of urbanization. On average, 82% of urban areas and 22.4% of rural areas in Sarawak are equipped with this service [34]. It is believed that only densely populated areas have the demand for such services.

Humans are the vectors spreading airborne infectious diseases, and population density reflects how closely people are packed together. Therefore, the effect of population density on COVID-19 spreading in a country or region becomes very influential after the intrusion or importation period of the epidemic through the areas with major transportation hubs [60]. For example, several previous studies presented evidence of the association between population density at the district level in Malaysia and the cumulative cases (or incidence rates) for the early outbreak until the Delta dominant period in the third quarter of 2021 [61–65]. Likewise, the effects of population density on Sarawak incidence rates are positive for all models in this study across different periods in 2021, except for Wave 3 in SEM. This corresponds to the aforementioned study [65], which highlighted that more populous and densely populated districts have a higher risk of transmission. Having said that, the local coefficients estimated for population density in GWR show mixed results across different districts, ascertaining the heterogeneity of the influence of the population density on district-wise incidence rates.

Another variable that is more inclined to give a positive relationship is the male population (i.e., the percentage of male residents). The coefficients estimated are consistently positive, except for Wave 1 in SLM and all GWR models. The finding agrees with an earlier study in which the infection rate was more among male and working-age people in Bangladesh in 2020 [66]. From the epidemiological variations perspective, the male population is more susceptible than the female population [67]. Although males and females had similar trends and rates of weekly cases in Kansas City, USA, in 2020, the clusters of the male population were more widely scattered than the female population [68].

The effect of piped water (i.e., percentage of households with piped water supply in the house), as well as the population who are 65 years and older on COVID-19 incidence rates, can be positive at one period and negative at another. The regression coefficients of Piped_water (resp. Pop_65) are positive (resp. negative) on all temporal periods, except for Wave 1 and Wave 2. It means that in the periods of Wave 1 and Wave 2, the findings of Piped_water agree with one study in Brazil in 2020 [59]. Undeniably, insufficient or unreliable water supply can aggravate poor sanitation, discourage hygiene practices and spread diseases in densely populated areas [69]. On the other hand, the opposite effects of Pop_65 in different temporal periods might suggest that the elderly population is probably more willing to adhere to preventive measures after they become aware of the seriousness of the COVID-19 situation in Sarawak in the first half of 2021.

Meanwhile, three other explanatory variables, namely, Public_h, Pop_15to64, and Pop_Growth, show counterintuitive results. They are all negatively affecting disease incidence rates across different models and waves. Among these, Public_h (i.e., the percentage of households with less than 5 km distance from living quarters to the nearest public health centers) is the most significant negative indicator. This might suggest that the spatial accessibility to medical services does not lead to more PCR (polymerase chain reaction) testing and more cases detected subsequently. This could be partly because local public health authorities intensively conducted targeted community-based swabbing activities and PCR testing on residents within localities declared for undergoing Enhanced Movement Order Control, rather than residents self-approaching health centers nearby for testing purposes.

The percentage of residents who are non citizens (Pop_NonCitizen) is an indicator not considered before in the ecological studies conducted for Malaysia. However, this indicator may be related to race, minority status, and language, which belong to one important domain of social vulnerability categorized in [37]. The non-citizen population is a minority in Sarawak, whereby only 10 out of 40 districts have a non-citizen population exceeding 10%. Yet, they are vulnerable since they may have access to fewer medical resources and are generally of lower socio-economic status. These may lead to a higher prevalence of COVID-19 in their community, as in other countries. For instance, nearly 95% of the laboratory-confirmed cases in Singapore in the first 8 months of 2020 were contributed by migrant workers with high-density and unhygienic living conditions [70]. Moreover, the share of a non-citizen from the total population, the non-English speaking population and high overseas migration turnover are categorized as the second principal component, which can explain the 23% variance of social vulnerability associated with COVID-19 in Australia [71]. However, our finding shows that the proportion of the non-citizen population is the driving force of the incidence rate only for Wave 1 (in global spatial models) and in some districts (in which their local GWR estimated coefficients in Waves 1, 2, and 4 are positive).

### Limitations and opportunities

As COVID-19 is a directly transmitted respiratory infection, human mobility plays an important role in its spatial distribution [72], either on a global or local scale. Although the inter-regional mobility flow can be indicated by the mobility data obtained through public transportation systems, mobile network operators, or mobile applications [15, 73], these technological data are scarce in developing regions like Sarawak. If such information was obtainable, the regression models in this study could have a better fitting.

Although the co-evolution of COVID-19 in the district level of Sarawak throughout 2021 is subdivided into four temporal periods, Wave 4 classified in this study (see Fig. 4) does not exhibit an increasing trend, followed by a peak and finally a decline to be considered as a pandemic wave. This is mainly becuase the district-wise confirmed case data are not continuously being published daily by SDMC at the beginning of the year 2022. If such data are available, then the observations in this study may alter. The peak of the Omicron wave is believed to occur in March 2022 in Sarawak on which the whole of Malaysia is already entering the last phase of the National Recovery Plan. During this phase, the majority of the confirmed cases are believed to be obtained from self-initiative tests using Antigen Rapid Test Kit, rather than PCR tests widely conducted in 2021.

Although the COVID-19 mass vaccination for the general public started in Sarawak in April 2021, the district-wise vaccination rates are not considered an explanatory variable in spatial regression models in this study. This is because the vaccination administration centers set up in one district in Sarawak do not necessarily provide injection services to the residents living in that district only. As a result, quite several districts recorded vaccination rates exceeding 100% of their population size based on our preprocessing work on the vaccination data publicly available and provided by the Ministry of Health Malaysia. If the vaccination data could represent the district-wise population-level vaccine-induced immunity, then the accuracy of the spatial regression models might be improved.

Apart from the two orders of contiguity-based spatial weights in this study, the use of other neighborhood matrices also need to be addressed when conducting spatiotemporal analysis [74]. Also, other GWR model choices such as multiscale GWR or mixed GWR should be explored [75]. Additionally, the temporal dependencies across different pandemic waves may be better comprehended through the Bayesian modelling framework [76]. Algorithm-based predictive modelling, which delves into the realm of machine learning and its methodologies can be valuable tools for analyzing COVID-19 data and making accurate predictions. All of these help improve the model fit in this study.

## Conclusion

This study provides a comprehensive analysis of the spatiotemporal variation of district-level COVID-19 incidence rates and their real scenario of relationship on socio-demographic factors in Sarawak, Malaysia. Our findings reveal significant spatial clustering patterns in different temporal periods. Specifically, we observe that the percentage of households with garbage collection facilities, population density, and the proportion of males in the population consistently exhibit positive associations with the increase of COVID-19 incidence rates across various model settings.

By shedding light on these relationships, our spatial modelling study offers valuable insights for local governments and public health authorities. It emphasizes the critical importance of integrating socio-demographic determinants of local communities into evidence-based decision-making for reshaping disease surveillance and response strategies. With a better understanding of the spatial patterns and socio-demographic factors influencing COVID-19 transmission, policymakers can make informed judgments and implement targeted interventions to effectively control the spread of the disease.

## Data Availability

The datasets used and/or analyzed during the current study are available from the corresponding author on reasonable request.

## Abbreviations

COVID-19: Coronavirus disease 2019
CV-E: Cross-validated error
OLS: Ordinary least squares
SLM: Spatial lag model
SEM: Spatial error model
GWR: Geographically weighted regression
WHO: World Health Organization
SDMC: Sarawak Disaster Management Committee
SOPs: Standard operating procedures
GIS: Geographic information system
Pop_GrowthR: Population growth rate
Pop_NonCitizen: Non-citizen population
Pop_Male: Male population
Pop_15to64: Population between 15 and 64
Pop_65: Population 65 years and above
Pop_Density: Population density
Median_inc: Median of monthly income
Public_h: Public health centers
LISA: Local indicators of spatial association
RMSE: Root mean square error
VIF: Variance inflation factor
AIC: Akaike information criteria
LR: Likelihood ratio
LM: Lagrange multiplier
PCR: Polymerase chain reaction
